# Bidirectional Attention for Text-Dependent Speaker Verification

**DOI:** 10.3390/s20236784

**Published:** 2020-11-27

**Authors:** Xin Fang, Tian Gao, Liang Zou, Zhenhua Ling

**Affiliations:** 1 School of Information Science and Technology, University of Science and Technology of China, Hefei 230022, China; klg@mail.ustc.edu.cn (X.F.); tiangao5@iflytek.com ( T.G.); zhling@ustc.edu.cn (Z.L.); 2 iFLYTEK Research, iFLYTEK Co., Ltd., Hefei 230088, China; 3 School of Information and Electrical Control Engineering, China University of Mining and Technology, Xuzhou 221116, China; 4 School of Electronics and Information Engineering, Anhui University, Hefei 236601, China

**Keywords:** text-dependent speaker verification, interactive representation, bidirectional attention, CNN

## Abstract

Automatic speaker verification provides a flexible and effective way for biometric authentication. Previous deep learning-based methods have demonstrated promising results, whereas a few problems still require better solutions. In prior works examining speaker discriminative neural networks, the speaker representation of the target speaker is regarded as a fixed one when comparing with utterances from different speakers, and the joint information between enrollment and evaluation utterances is ignored. In this paper, we propose to combine CNN-based feature learning with a bidirectional attention mechanism to achieve better performance with only one enrollment utterance. The evaluation-enrollment joint information is exploited to provide interactive features through bidirectional attention. In addition, we introduce one individual cost function to identify the phonetic contents, which contributes to calculating the attention score more specifically. These interactive features are complementary to the constant ones, which are extracted from individual speakers separately and do not vary with the evaluation utterances. The proposed method archived a competitive equal error rate of 6.26% on the internal “DAN DAN NI HAO” benchmark dataset with 1250 utterances and outperformed various baseline methods, including the traditional i-vector/PLDA, d-vector, self-attention, and sequence-to-sequence attention models.

## 1. Introduction

Automatic speaker verification (SV) aims to verify the identity of a person based on his/her voice. It can be categorized into text-dependent and text-independent types, according to whether the lexicon content of the enrollment utterance is the same as that of evaluation utterance [1,2,3,4]. In general, the text-dependent SV (TDSV) outperforms the text-independent type due to its phonetic variability and robust handling of short utterances [5,6]. Especially with the development of smartphone and mobile applications, interacting with mobile devices through a short speech is becoming more and more popular, and voice authentication through a given speech password has been widely accepted [7]. In this study, we focus on TDSV with the global password “DAN DAN NI HAO” (in Chinese), which is used as the wake-up voice for the Alpha Egg product of iFlytek.

Typically, similar to other classification tasks in machine learning, the pipeline of speaker verification includes feature extraction, modeling and classification strategy. To be more specific, various frame-level acoustic features, such as Mel-frequency cepstral coefficients (MFCC) and power normalized cepstral coefficients (PNCC), are widely employed as front-end features [8]. Then the Gaussian mixture model–universal background model (GMM-UBM) or the i-vector strategy can be utilized for speaker modeling [9]. At the last stage, a probabilistic linear discriminant analysis (PLDA) or a simple cosine distance is usually employed to calculate the similarity between the representations of enrollment and evaluation utterances [10].

Prior to the development of deep learning, i-vector in tandem with PLDA were the dominating approach of SV [1,11]. Benefitting from the nonlinear representation ability of neural networks, deep learning-based methods have shown promising results in both text-independent SV and TDSV [12,13,14]. Deep neural networks (DNN) are employed to either extract frame-level speech features or replace the traditional GMM-UBM for partitioning the feature space [10]. For instance, Garcia-Romero et al. proposed a DNN-based approach to compute the alignments and the speaker features for statistics [15]. Liu et al. investigated four types of deep learning models for extracting deep features, which were further analyzed via an i-vector-based framework [10]. The extracted frame-level features are always equally weighted and averaged into utterance-level speaker representation (i.e., d-vector). However, it was shown that the averaging operation might ignore the content information and therefore deteriorate the performance [16].

Recently, the end-to-end approaches have become more preferable in text-dependent speaker verification [3,16]. The biggest advantage of end-to-end methods is that all model parameters can be simultaneously optimized based on one loss function. To the best of our knowledge, Google was the first to propose the end-to-end method for training DNNs in TDSV [3]. Compared with the previous approaches to TDSV, they constructed the discriminative model directly from utterances. In addition, the corresponding model was more compact and showed better performance [3]. They demonstrated the effectiveness of the proposed model on the internal “OK Google” benchmark dataset. Instead of treating the frame-level features equally, researchers at Microsoft introduced an attention mechanism and combined the frame-level features into utterance-level features [17]. Inspired by the success of convolution neural networks (CNNs) in many speech recognition problems, they extracted noise-robust features via speaker discriminative CNNs. They further demonstrated the performance enhancement on Window 10’s “Hey Cortana” dataset. End-to-end strategies seem more promising to achieve better performance than the classical i-vector-based systems in TDSV.

The attention mechanism method has been widely employed and produced significant improvements in various tasks of TDSV, especially in the last three years [7,18,19]. It provides a powerful way to learn long-range dependencies and emphasize the most relevant information of the input utterances. Bian et al. proposed a novel strategy incorporating a residual network (ResNet) with the self-attention mechanism and achieved satisfying performance with fewer parameters and less computational cost [18,20]. However, the authors assumed that the enrollment speaker’s representation was constant and did not consider the influence of the evaluation utterances. More recently, Zhang et al., in Tencent AI Lab, proposed a single-directional sequence-to-sequence (Seq2Seq) attention-based method and generated an utterance-level enrollment evaluation joint vector to evaluate the similarity between the enrollment and evaluation utterances [7]. They showed that the proposed method outperformed many baseline models, including the classical i-vector/PLDA, d-vector method, and self-attention-based approach on the Tencent "9420" wake-up word dataset.

Despite the significant performance improvement that has been achieved via deep learning-based methods, there are still some issues that need to be tackled. The motivations of the proposed method are summarized as the following three aspects.

First and foremost, most of the existing methods assume that the target speaker representation (i.e., the features corresponding to enrollment utterance) is constant when comparing with different evaluation utterances. However, in human speaker verification, people tend to pay attention to different features of the enrollment utterance in comparison with various evaluation utterances. To the best of our knowledge, the research considering the effect of evaluation utterances on extracting the target speaker representation is still limited.

Second, most of the existing TDSV methods employ a metric loss function (e.g., triplet loss) to maximize the within-class similarity $s_{p}$ and minimize the between-class similarity $s_{n}$. However, these kinds of loss functions assume the penalty strengths on $s_{p}$ and $s_{n}$ equally, and seek to reduce ($s_{n}-s_{p}$). In some extreme cases, e.g., $s_{n}$ is large and $s_{p}$ already approaches 1, the methods keep on penalizing $s_{p}$ with a large gradient. Given one of the similarity scores deviates far from the optimum, it should receive a strong penalty. It was demonstrated that the optimization strategy of triplet loss lacks flexibility and might lead to irrational results [21]. In addition, optimizing ($s_{n}-s_{p}$) usually provides a decision boundary of $s_{p}-s_{n}=m$, multiple statuses on which are accepted as the convergence statuses. Consequently, the ambiguous convergence might deteriorate the classification performance [21].

Finally, although there were a few attempts to apply various attention mechanisms to TDSV, researchers tend to neglect the content information of the speech signal in training the attention model. It was shown that phonetic information can significantly improve the performance of TDSV.

To address the above-mentioned concerns, we propose a novel framework based on a bidirectional attention and convolution neural network (BaCNN) to generate dynamic speaker representations for both enrollment utterance and evaluation utterance and to verify the speaker’s identity effectively. The main contributions of the proposed method are threefold:

(1) For each pair of compared utterances (including one for enrollment and another for evaluation), attention scores for frame-level hidden features are calculated via a bidirectional attention model. The input of the model includes the frame-level hidden features of one utterance and the utterance-level hidden features from the other utterance. The interactive features for both utterances are simultaneously obtained in consideration of the joint information shared between them. To the best of the authors’ knowledge, we are the first to employ bidirectional attention in the speaker verification field.
(2) Inspired by the success of circle loss in image analysis, we replace the triplet loss in conventional TDSV models with the recently proposed circle loss. It dynamically adjusts the penalty strength on the within-class similarity and between-class similarity, and provides a flexible optimization. In addition, it tends to converge to a definite status and hence benefits the separability.
(3) We introduce one individual cost function to identify the phonetic contents, which contribute to calculating the attention score more specifically. The attention is then used to perform phonetic-specific pooling. Experimental results demonstrate that the proposed framework achieves the best performance compared with classical i-vector/PLDA, d-vector, and the single-directional attention models.

The rest of this paper is organized as follows. Section 2 demonstrates the state-of-the-art text-dependent speaker verification techniques. Section 3 introduces the proposed bidirectional attention mechanism and the detailed network settings. Section 4 shows the experimental setup and Section 5 presents the experimental results. Finally, Section 6 presents the conclusions.

## 2. State of the Art

To facilitate the comparison with our proposed bidirectional attention (i.e., evaluation-specific attention), we review the basics of a few state-of-the-art methods, including i-vector/PLDA, d-vector, naïve attention based TDSV.

### 2.1. TDSV Based on i-Vector

The i-vector based feature extractor was originally proposed by Dehak et al. and has become a popular strategy in TDSV [5,9]. Instead of defining two separate spaces as in joint factor analysis (JFA), the authors only defined the total variability space, which simultaneously contains the speaker and channel variabilities [22,23]. Given an utterance, the speaker- and channel-dependent supervector *M* is modeled as:

(1) M=m+Tw,

where *m* is the mean supervector of the universal background model (UBM), *T* is the total-variability matrix defining the total variability space, and *w* is a random vector following the standard normal distribution and representing the low-dimensional total-variability factors, i.e., i-vector. Each factor controls one separate eigen-dimension of *T*. Given one utterance, the corresponding i-vector is the maximum a posterior probability (MAP) estimation of *w*. We refer the interested readers to [22,23,24] for more details.

The total variability space includes the channel variability arising from phonetic and channel variations. In order to attenuate the disturbance from channel variability, various channel compensation techniques have been explored. For TDSV, PLDA is always employed as the back-end for modeling the within-speaker and between-speaker variability.

### 2.2. TDSV Based on d-Vector

Compared with the i-vector based on traditional spectral features (e.g., MFCC), the outputs from the hidden layer of various deep models, referred to as a d-vector, tend to provide better performance in TDSV [3,10,25]. Li et al. evaluated the performance of recurrent neural network (RNN)-based and CNN-based architectures in TDSV, and CNN was shown to be more powerful in modeling the acoustic features [26]. In this work, we employ the CNN-based architecture to extract the d-vector of each utterance and measure the cosine-distance between them.

**Figure 1:**
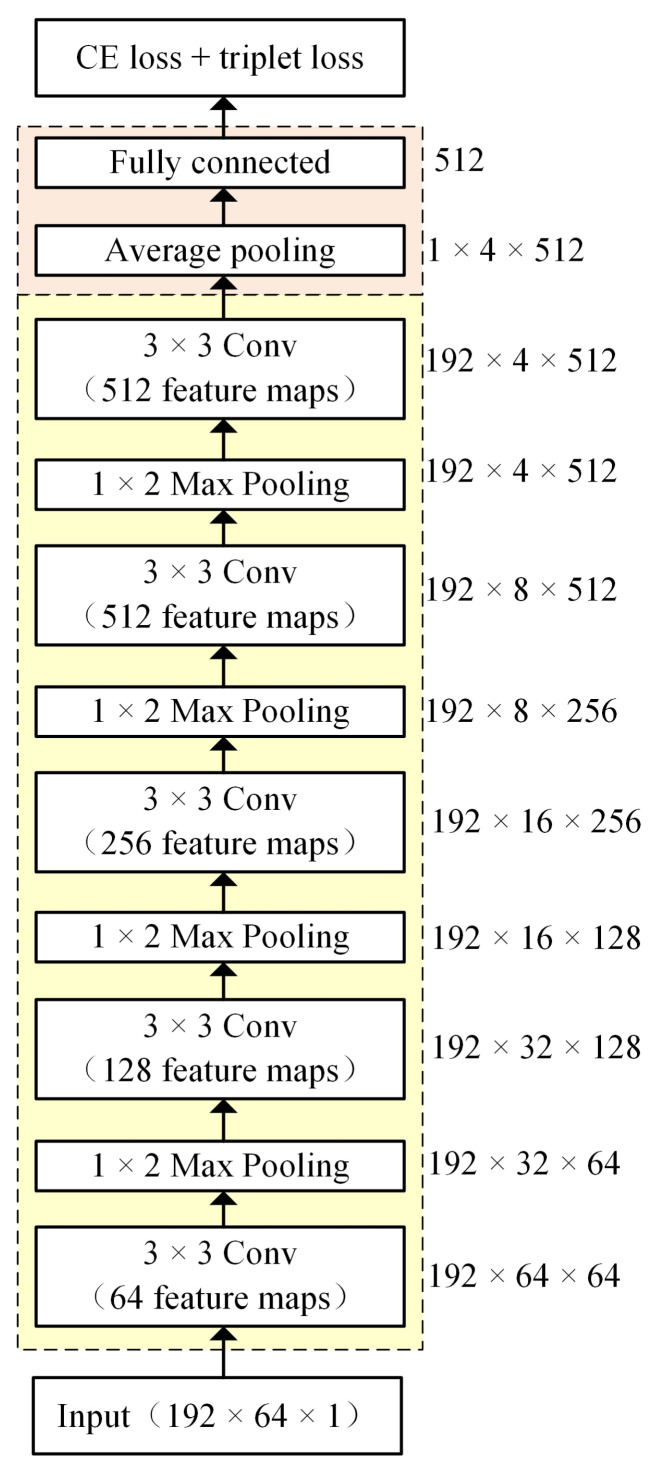
The architecture of convolutional neural network (CNN)-based d-vector extraction model. Cross-entropy (CE) loss and triplet loss are used in this study.

Figure 1 shows the topology of the baseline CNN for extracting the d-vector. The inputs include 192 frames of 64-dimensional filter-bank features. Each convolutional layer is followed by a pooling layer with 1×2 max pooling. The average pooling and the last fully connected layer are used to obtain the utterance representation. The total loss is a combination of the softmax cross entropy loss and the triplet loss [27]. The softmax cross entropy loss is defined as:

(2) $$L_{s}=-\sum_{i=1}^{M}log\left(\frac{e^{W_{y_{i}}^{T}x^{i}+b_{y_{i}}}}{\sum_{j=1}^{N}e^{W_{j}^{T}x^{i}+b_{j}}}\right),$$

where $x^{i}$ denotes the *i*-th speaker embedding, corresponding to the $y_{i}$ speaker. $w_{j}$ denotes the *j*-th weights vector and b is the bias term in the last fully connected layer. *M* and *N* represent the mini-batch size and the number of speakers, respectively. The triplet loss is defined as:

(3) $$L_{T}=\sum_{i=1}^{M}max\left(0,D\left(x^{i},x^{n}\right)+\delta -D\left(x^{i},x^{p}\right)\right).$$

The triplet loss is calculated via triplets of training samples ($x^{i},x^{n},x^{p}$), where ($x^{i},x^{p}$) belong to the same speaker and ($x^{i},x^{n}$) are from different speakers. Intuitively, the triplet loss minimizes the distances between utterances from the same speaker and maximizes the distance between utterances from different speakers.

### 2.3. TDSV Based on Attention Mechanism

**Figure 2:**
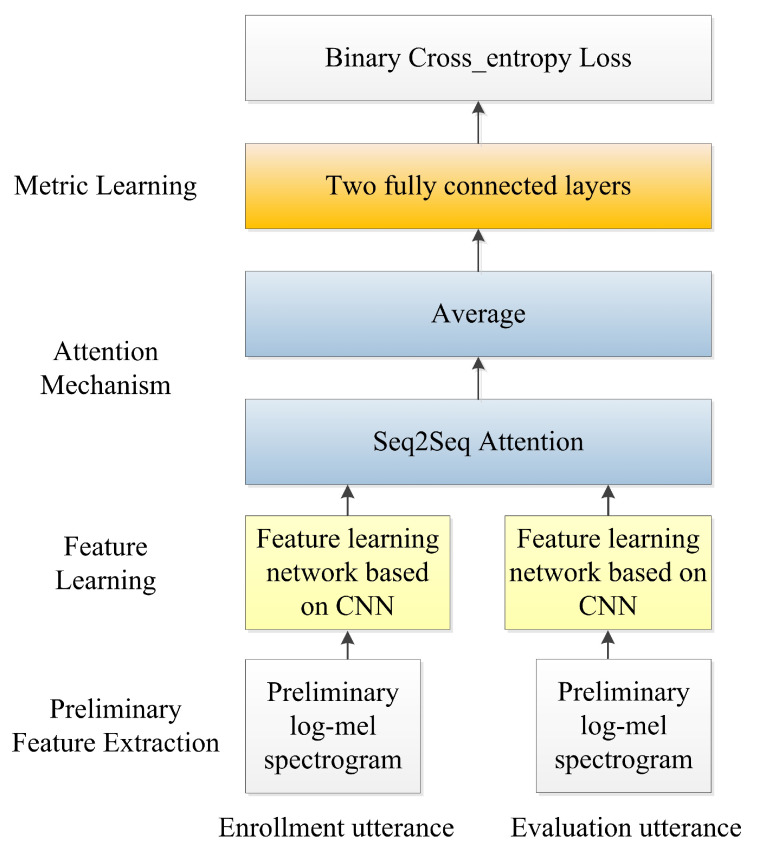
Demonstration of the Sequence to Sequence (Seq2Seq) [7] attention-based text-dependent speaker verification (TDSV) model.

Inspired by human attention behavior [28], a recent trend in TDSV is to build deep learning-based TDSV systems with attention mechanisms [7,19]. Most of the existing methods aim to combine the frame-level features via the combination weights learned from the attention model. These methods extract the utterance-level features for each utterance separately, and thus neglect the joint information between enrollment and evaluation utterances, as in [17,19]. To address this concern, Zhang et al. from Tencent AI Lab proposed a sequence-to-sequence attentional Siamese model, and generated an enrollment evaluation joint vector for each pair of enrollment and evaluation utterances [7]. The architecture of this TDSV model is shown in Figure 2, and includes feature learning, an attention mechanism, and metric learning. In the feature learning section, the model learns the frame-level features from the primary log-mel spectrogram. Then, the sequence-to-sequence model is used to compute the attention weights for the temporal alignment between the features obtained at the previous feature learning stage. Finally, at the metric learning stage, two fully connected layers, with 108 units and one unit, respectively, determine whether these two utterances are from the same speaker.

## 3. Methodology

**Figure 3:**
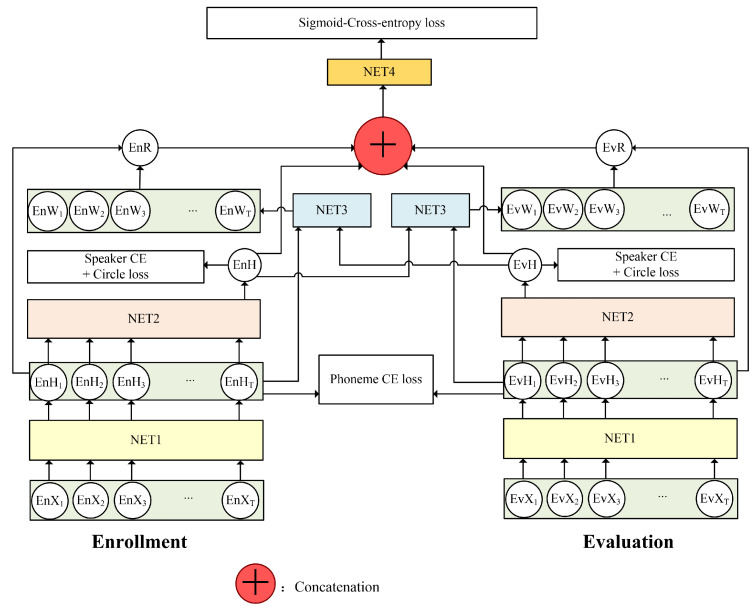
The architecture of the proposed bidirectional attention-based TDSV model, including NET1 for frame-level hidden feature extraction, NET2 for feature combination, NET3 for the bidirectional attention, and NET4 for the metric learning.

The human brain has selective auditory attention [29], allowing attention to be directed to different acoustic features of interest in various speech perception tasks. For the speaker verification task, human listeners tend to pay attention selectively when comparing each pair of utterances. Given different pairs of utterances, people are able to change their attention according to the joint information between the enrollment and evaluation utterances. Different distinguishable features should be focused on when verifying various samples. In this paper, we propose a novel bidirectional attention mechanism to mimic the human auditory attention system by learning an interactive and speaker-discriminative feature representation. Inspired by the success of CNN in TDSV, we employ the CNN-based architecture (NET1 in Figure 3) as described in Section 2.2 to extract the frame-level features $EnH_{t}$ and $EvH_{t}$ for the *t*-th frame of enrollment and evaluation utterances, respectively. These frame-level features are further aggregated into utterance-level ones—EnH and EvH—via the NET2. The bidirectional attention model then computes the attention weights of each of the frame-level features of one utterance based on the joint information with the utterance-level features of the other utterance, and obtains the interactive speaker representations of these two utterances. Finally, the last fully connected layer NET4 predicts whether these two utterances belong to the same speaker based on the combination of the interactive representations (i.e., EnR and EvR) and constant utterance-level hidden features (i.e., EnH and EvH).

### 3.1. Data Preprocessing

Considering the difference of speech length, we apply zero-padding or truncating to obtain the fixed length of 192 frames. Specifically, if the duration of an utterance is shorter than 192 frames, we use zero-padding at the beginning of the utterance. Otherwise, we take the first 192 frames of this utterance, and the rest of the speech is discarded. This rarely happens since the durations of four words usually do not exceed 192 frames. A masking mechanism is utilized to eliminate the effect of zero-padding in the training process.

### 3.2. Model Structure

As stated above, NET1, used for frame-level feature extraction and NET2, used for feature combination, have the same topology as the one shown in Figure 1. However, differently from the CNN-based d-vector extraction model, the proposed method takes a pair of inputs and has two branches, NET1 and NET2, which share weights. NET1 includes five convolutional layers. Each of the first four convolutional layers is followed by a max pooling layer. In this study, we empirically set the convolution kernel size as 3 × 3 and filter size as 1 × 2. The NET2 includes one average pooling layer and one fully connected layer, which is the same as the green part shown in Figure 1.

**Figure 4:**
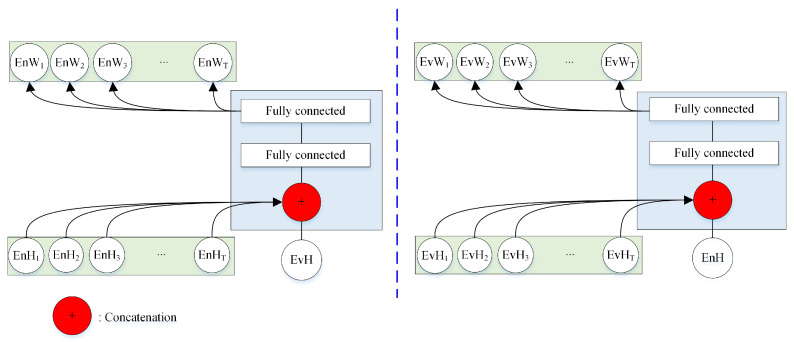
The structure of the bidirectional attention mechanism. For either branch, the frame-level hidden features of one utterance and the utterance-level hidden features of the other utterance are adopted as the inputs.

Take the left branch for instance: the outputs of NET1, the frame-level hidden features, are denoted as:

(4) $$\begin{array}{c} \left(EnH_{1},EnH_{2},\ldots ,EnH_{T}\right)\\ =f_{Net1}\left(EnX_{1},EnX_{2},\ldots ,EnX_{T},\theta_{NET1}\right), \end{array}$$

where $EnX_{t}$ is the *t*-th frame of the enrollment utterance and $EnX_{t}$ is the corresponding frame-level speech feature, $\theta_{NET1}$ represents the parameters of NET1. These frame-level hidden features are further analyzed by NET2, including one average pooling layer and one fully connected layer. The utterance-level hidden features EnH are obtained as:

(5) $$EnH=f_{NET2}\left(EnH_{1},EnH_{2},\ldots ,EnH_{T},\theta_{NET2}\right),$$

where $\theta_{NET2}$ represents the parameters of NET2. Traditionally, the output of NET1 and NET2 is either directly taken as the speaker embedding, or the attention weights for one utterance are calculated based on the information from itself. In this study, we developed a bidirectional attention model (NET3 in Figure 3) to capture the joint information between the enrollment and evaluation utterances. For instance, the left branch, NET3, takes all of the frame-level hidden features of enrollment utterance and the utterance-level hidden features of the evaluation utterance, and outputs the attention weights of these frame-level features $EnW_{t}$, as shown in Figure 4. NET4 includes two fully connected layers. The attention weights are obtained as follows:

(6) $$\begin{array}{c} \left(EnW_{1},EnW_{2},\ldots ,EnW_{T}\right)\\ =f_{NET3}\left(EnH_{1},EnH_{2},\ldots ,EnH_{T},EvH,\theta_{NET3}\right), \end{array}$$

(7) $$\begin{array}{c} \left(EvW_{1},EvW_{2},\ldots ,EvW_{T}\right)\\ =f_{NET3}\left(EvH_{1},EvH_{2},\ldots ,EvH_{T},EmH,\theta_{NET3}\right), \end{array}$$

where $\theta_{NET3}$ represents the parameters of NET3. The attention weights for either utterance are obtained in view of the joint information between two utterances.

Finally, we employ a discriminator NET4 with one fully connected layer to decide whether these two utterances belong to the same speaker. The decision, D, is made according to:

(8) $$D=f_{NET4}\left(EnR,EvR,\theta_{NET4}\right),$$

where $\theta_{NET4}$ represents the parameters of NET4. EnR and EvR denote the interactive speaker representations corresponding to the enrollment utterance and evaluation utterance, respectively. They are the weighted sums of the frame-level hidden features, as per the following:

(9) $$EnR=EnW_{t}\times EnH_{t},$$

(10) $$EvR=EvW_{t}\times EvH_{t}.$$

The parameters of these four parts, including NET1 and NET2 for feature extraction, NET3 for calculating the attention weights, and NET4 for the final metric learning, are jointly optimized via end-to-end training.

### 3.3. End-to-End Training

In TDSV, we should consider the discriminative information from both the speakers and the text. The end-to-end loss in this study is a combination of the losses from NET1, NET2, and NET4, considering speaker-discriminant and text-discriminant factors. The phoneme softmax cross-entropy loss of NET1 is defined as:

(11) $$L_{NET1}=-\sum_{i=1}^{M}\sum_{t=1}^{T}log(\frac{e^{W_{y_{i}}^{T}H_{t}^{i}+b_{y_{i}}}}{\sum_{j=1}^{N}e^{W_{j}^{T}H_{t}^{i}+b_{j}}}),$$

where $H_{t}^{i}$ denotes the *t*-th frame-level hidden feature of the *i*-th utterance, belonging to the $y_{i}$ phoneme. $W_{j}$ denotes the *j*-th column of the weight matrix *W* in the last fully connected layer and *b* is the bias term. *M* is the size of the mini-batch and *N* is the number of phonemes in each utterance. *T* is the length of each utterance. This loss for phoneme classification contributes to avoiding the misalignment of frame-level features for the convolution and pooling operations, and enables the network to fasten its attention on the features of interest specifically.

The NET2 loss is a mixed loss with a combination of the softmax cross-entropy loss and the circle loss, and is defined as follows:

(12) $$L_{NET2}=L_{S}+L_{C},$$

where $L_{s}$ is the softmax cross-entropy (CE) loss and and $L_{C}$ is the circle loss defined as follows:

(13) $$L_{C}=\log\left[1+\exp\left(\theta_{n}\left(S_{n}-\Delta_{n}\right)\right)\times \exp\left(-\theta_{p}\left(S_{p}-\Delta_{p}\right)\right)\right.,$$

where $\theta_{n}$ and $\theta_{p}$ are nonnegative weighting factors, and $\Delta_{n}$ and $\Delta_{p}$ are the between-class and within-class margins, respectively. For detailed calculation of these parameters, please refer to [21]. Differently from the triplet loss in conventional TDSV, $s_{n}$ and $s_{p}$ are in an asymmetric position. The circle loss dynamically changes the penalty strength and hence is able to provide a more balanced optimization on these two similarities.

The NET4 loss is the sigmoid cross-entropy loss, which is defined as follows:

(14) $$L_{NET4}=\delta \left(j,k\right)\sigma \left(S\right)+\left(1-\delta \left(j,k\right)\right)\left(1-\sigma \left(S\right)\right),$$

where $\sigma \left(S\right)=1/\left(1+e^{-S}\right)$ is the standard sigmoid function. δ(j,k) equals 1 when j=k; otherwise it equals 0.

We trained the overall network based on a two-step strategy, including the first step to pre-train NET1 and NET2 for effective speaker representations and the second step for jointly training all four NETs. The overall network is optimized via the stochastic gradient descent (SGD) approach [30]. The optimization formulas can be written as:

(15) $$\theta_{NET1}=\theta_{NET1}-l\times \left(\gamma \times \frac{\partial L_{NET1}}{\partial \theta_{NET1}}+\frac{\partial L_{NET2}}{\partial \theta_{NET1}}+\beta \times \frac{\partial L_{NET4}}{\partial \theta_{NET1}}\right),$$

(16) $$\theta_{NET2}=\theta_{NET2}-l\times \left(\frac{\partial L_{NET2}}{\partial \theta_{NET2}}+\beta \times \frac{\partial L_{NET4}}{\partial \theta_{NET2}}\right),$$

(17) $$\theta_{NET3}=\theta_{NET3}-l\times \left(\frac{\partial L_{NET4}}{\partial \theta_{NET3}}\right),$$

(18) $$\theta_{NET4}=\theta_{NET4}-l\times \left(\frac{\partial L_{NET4}}{\partial \theta_{NET4}}\right),$$

where *l* is the learning rate, and γ and β are the weights corresponding to the losses of NET1 and NET4, respectively.

For each neural network model, an SGD optimizer with a momentum of 0.9 was employed. We set the learning rate to be 0.1024 in the first five epochs and decreased it to 0.05012 in the second five epochs. Each combination of neural network architecture and loss function was trained for 10 epochs in total. Additionally, we employed the batch to accelerate the training process. Once the network is trained, the enrollment speech and evaluation speech can be sent to the model simultaneously to verify whether the evaluation speech is also from the speaker who provides the enrollment speech.

## 4. Experimental Setup

### 4.1. Experimental Dataset

We evaluated the proposed bidirectional attention-based TDSV system on our internal “Dan Dan Ni Hao” benchmark dataset. The dataset includes three subsets for training, development (i.e., validation), and testing. The sampling rate is 16 kHz and the precision is 16-bit. All of these audio recordings were collected from three homemade sessions at iFlytek Co., Ltd. These utterances were forced aligned to obtain the “Dan Dan Ni Hao” snippets. There are around 120 frames for each snippet, with a frame rate of 100 Hz. In view of this observation, we extracted the first 120 frames from each snippet. For the snippet with less than 120 frames, we padded frames with zeros at the end.

Training Set: This set included 6900 speakers, and each speaker has 77 utterances on average. Utterance duration is 1.2 s on average.

Development Set: All utterances from the other 25 speakers were used as a validation set for adjusting the hyperparameters. For each speaker, one utterance was used as the enrollment data and the other 25 utterances were used for evaluation. This resulted in 625 target trials and 15,000 impostor trials in total.

Test Set: The test set comprised recordings from 50 speakers. For each speaker, one recording was used for enrollment, and the other 25 were used as evaluation utterances. This resulted in 1250 target trials and 30,000 impostor trials in total. The test set did not have any overlap with the training set or the development set, in terms of speakers.

### 4.2. Evaluation Metric

To fairly evaluate the performance of the proposed method, we employed three performance indices, including equal error rate (EER), Recall, and Minimum of detection cost function (MinDCF) [31,32]. EER is determined when the false alarm (false acceptance) probability equals to the miss (false rejection) probability. The lower the EER value, the higher the accuracy of the TDSV system. Recall is a measure of true positive rate, defined as:

(19) $$Recall\left(\theta \right)=\frac{TP}{TP+FN\prime }$$

where TP represents the number of true positive samples, FN represents the false negative samples (missed detections), and θ represents the verfication threshold. In this work, we evaluated the Recall when the false alarm rate equals 0.05, denoted as $Recall_{0.05}$. DCF is defined as a weighted sum of the miss and false alarm probabilities:

(20) $$DCF\left(\theta \right)=C_{Miss}\times P_{Target}\times P_{Miss}\left(\theta \right)+C_{FalseAlarm}\times \left(1-P_{Target}\right)\times P_{FalseAlarm}\left(\theta \right),$$

where $P_{Miss}\left(\theta \right)$ and $P_{FalseAlarm}\left(\theta \right)$ represent the miss and the false alarm probabilities, respectively. $C_{Miss}$ and $C_{FalseAlarm}$ denote the relative cost of false rejection and false acceptance, respectively, and were empirically set to be 10 and 1, respectively, as in [33]. $P_{Target}$ is the a priori probability of the specified target speaker. We evaluated the DCF when the $P_{Target}=0.01$, namely $DCF_{0.01}$. The $DCF_{0.01}$ varies with different verification thresholds (θ), and we evaluated the verification performance with the minimum value of $DCF_{0.01}$ (i.e., $MinDCF_{0.01}$). Furthermore, below we show the performance of the detection error trade-off (DET) curves, which demonstrate the error at different operating points.

## 5. Results and Discussion

To fully explore the effectiveness of the proposed bidirectional attention-based TDSV system, experiments and comparisons in terms of the network architecture, hyperparameters, and different attention mechanisms were designed.

### 5.1. The Weights of Losses

**Figure 5:**
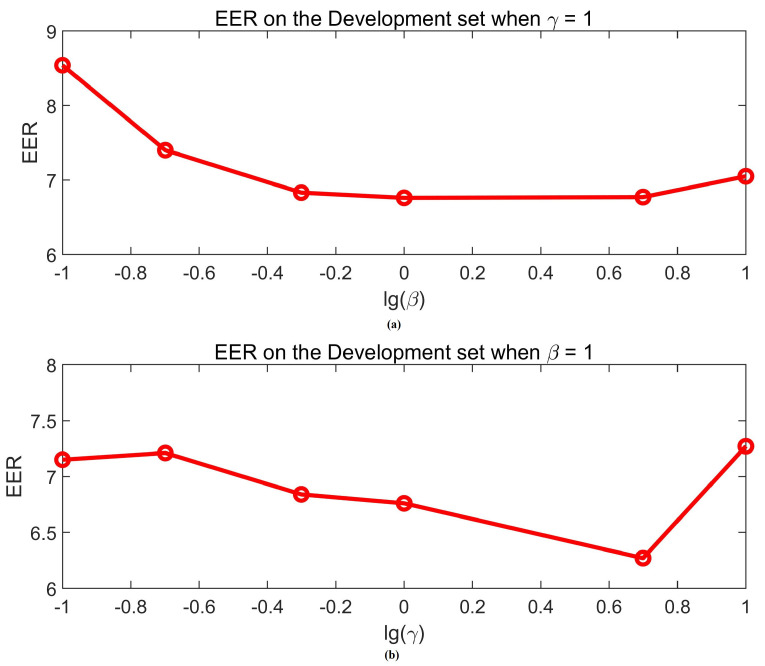
The equal error rate (EER) corresponding to different weights of losses. (**a**) the EER on the development set when γ=1; (**b**) the EER on the development set when β=1.

We investigated the performance of models with different loss weights. We utilized two hyperparameters to achieve a tradeoff between the losses of NET1, NET2, and NET4, including γ as the weight for the loss of NET1 and β as the weight for the loss of NET4. Considering both hyperparameters might affect the performance, we changed one parameter at a time. We heuristically initialized the γ as 1, and the impact of β on EER and on the development set are depicted in Figure 5a. The lowest EER of 6.76% on the development set was achieved when β was set to 1. We further evaluated the impact of γ when β equals the potential optimal value 1. As shown in Figure 5b, the proposed method achieved the potential lowest EER of 6.27% on the development set when γ was set to 5. For simplicity, we set γ to 5 and β to 1 in following experiments.

The TDSV system should focus not only on the speakers’ discriminative features but also on phonetic information. Therefore, we introduced the softmax cross-entropy loss of NET1, focusing on the lexical contents of each frame. Because of the multi-layer convolution and pooling operations in NET1, the *t*-th frame-level hidden feature without $L_{NET1}$ may lack information with regard to the lexical contents. To make the attention mechanism more specific, we employed the force alignment strategy to obtain the phoneme label for each frame of speech. In this study, we also evaluated the performance without the $L_{NET1}$, and the performance is listed in Table 1. The EERs considering the phonetic information were 6.27% and 6.26% on the development and test sets, respectively. In contrast to the EER neglecting the phonetic contents, it achieved a relative decrease of 5.0% and 5.44% on the development and test sets, respectively. This indicates the importance of the temporal alignment within each pair of enrollment and evaluation utterances.

### 5.2. Comparison between Triplet Loss and Circle Loss

We compared the circle loss against the triplet loss, which was commonly used in TDSV systems. We comprehensively evaluated their performance in both the traditional d-vector-based architecture and the proposed BaCNN framework, where the circle loss or triplet loss are combined with the cross-entropy loss. As listed in Table 2, the circle loss outperformed the triplet loss. More specifically, as for the traditional d-vector-based framework shown in Figure 1, the combination of CE loss and circle loss achieved an EER of 7.43% and 7.18% on the development and test sets, respectively, a relative decrease of 7.47% and 10.14%, respectively, compared to the combination of CE loss and triplet loss. In addition, as for the proposed bidirectional attention framework, either combination with circle loss or triplet loss were used as the loss function for the NET2. The optimal weights for $L_{NET1}$ and $L_{NET4}$ were selected with the strategy mentioned in Section 5.1. The BaCNN model with circle loss consistently outperformed that with the triplet loss on both the development set and test set. We suspect that the improvement is mainly due to the better separability in the feature space learned by the circle loss. In addition, the circle loss benefits deep feature learning with high flexibility. Considering the distances to the optimum, the circle loss assigns different gradients to these similarity scores, rather than as that in the triplet loss, where the within-class similarity and between-class similarity are in symmetric position.

### 5.3. Evaluation of Different Deep Feature Combinations

Utterance-level hidden vectors, EnH and EvH, are derived from deep convolutional network separately, and are employed as the constant d-vectors to represent the speaker identity [26]. However, these d-vectors do not consider the joint-information between enrollment and evaluation utterances. In this work, we employ a bidirectional attention model to mimic humans’ selective auditory attention [28,29]. For each pair of compared utterances, we extract the interactive speaker representation of either utterance in consideration of the information from the other one, and obtain the corresponding features for speaker verification, EnR and EvR. We evaluated the performance obtained when using features at different levels, including the utterance-level hidden features EnH and EvH, which are constant, the speaker representations via bidirectional attention, EnR and EvR, which are interactive, and their combination. As shown in Table 3, the combination of these two kinds of features provided the best performance. Compared with the traditional method based on utterance-level hidden features, the proposed method achieved a 4.27% and 5.44% relative decrease of EER on the development set and test set, respectively. In addition, we evaluated the performance of unidirectional attention, where the speaker representation of either enrollment utterance or evaluation utterance is assumed to be constant. The combination of EnH, EvH, and EnR provided comparable performance on the development set, whereas it showed higher EER on the test set. This performance suggests that the joint information between two utterances is complementary to the traditional d-vector.

### 5.4. Comparison with State-of-the-Art TDSV Methods

In this experiment, we compare our proposed method with several state-of-the-art text-dependent speaker verification methods, including the methods based on i-vector, d-vector, self-attention, and Seq2Seq attention, in terms of the EER, $Recall_{0.05}$, and $MinDCF_{0.01}$. As for the i-vector extraction, a UBM with 512 Gaussian mixture components was used to collect the Baum–Welch statistics from the training utterances, and a gender-independent total variability matrix with 300 total factors was obtained. We further employed the LDA and within-class covariance normalization (WCCN) to alleviate intra-speaker variability and reduce the dimension of the i-vector to 200 [12,24]. A PLDA model with 150 latent identity factors was then trained. In this study, we employed the CNN architecture detailed in Section 2.2 to extract the frame-level features. As for the d-vector-based strategy, the cosine distance was used to evaluate the similarity between the speaker representations obtained from enrollment and evaluation utterances separately. Instead of averaging, we also evaluated the performance of the self-attention and Seq2Seq attention mechanism as in [7,19], which are used to calculate the weights of frame-level hidden features. It should be mentioned that the utterance-level feature learning modules of these deep learning-based frameworks were pre-trained based on the entire training set.

Except for the traditional i-vector based strategy, the other methods employ the same basic speaker representations as the d-vector-based method. As can be seen in Table 3, introducing attention mechanisms into TDSV models improved the verification performance in various tasks. In addition, considering the joint information between enrollment and evaluation utterances, the proposed BaCNN approach achieved the best performance, as listed in Table 4. Compared with the d-vector baseline system, the proposed method achieved a relative decrease of 15.61% and 12.81%, a relative increase of 4.94% and 2.24%, and a relative decrease of 8.03% and 0.52%, in terms of the EER, $Recall_{0.05}$, and $MinDCF_{0.01}$, respectively. The result on the test set is consistent with that on the development dataset, which further shows the robustness of the proposed BaCNN strategy.

**Figure 6:**
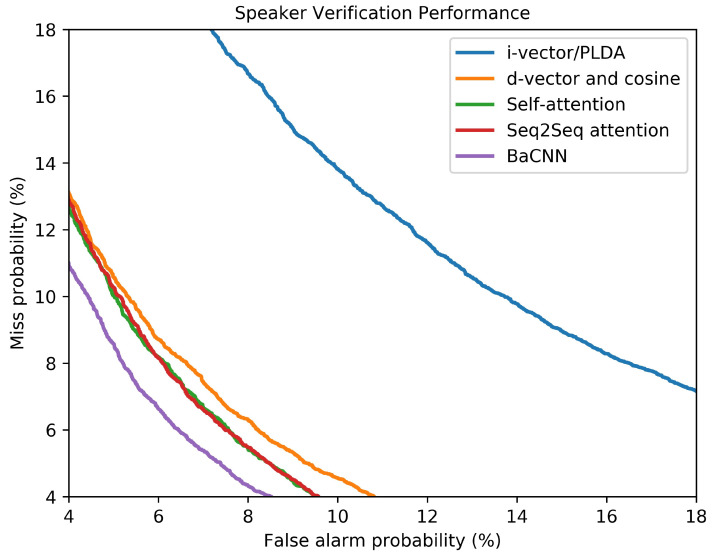
DET curves of different methods.

If all the parameters of these four NETs are randomly initialized and jointly optimized (denoted as BaCNN-1step), the performance is not competitive. At the early stage of the training, it is difficult to provide effective attention due to the inaccurate constant speaker representations (i.e., EnH and EvH). Therefore, the model cannot quickly converge to a relatively good solution. After pre-training of NET1 and NET2, the joint training of NET3 and NET4 provided better performance. The corresponding EER had a relative decrease of 17.5% and 9.41% compared to that of the BaCNN-1step on the development set and test set, respectively. In addition, the DET curves in Figure 6 show a comparison with the state-of-the-art TDSV methods mentioned above, and further demonstrate that the improvements were consistent across operating points.

### 5.5. Analysis of Interactive Speaker Embeddings

**Figure 7:**
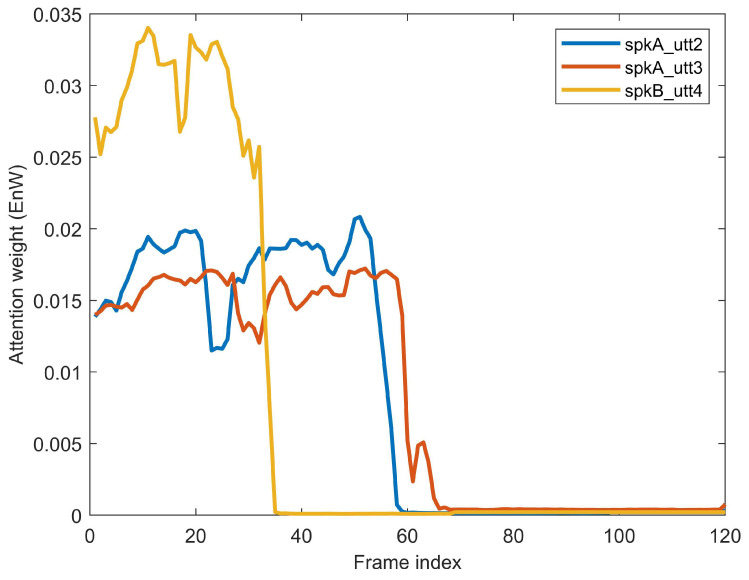
An illustrative example of the attention weight EnW. The physical length of the enrollment Spk_utt1 is 120 frames.

In order to illustrate the effectiveness of the proposed bidirectional attention mechanism, we further analyzed the attention weights corresponding to different pairs of enrollment and evaluation utterances. We randomly selected utterance1 of speaker A (denoted as SpkA_utt1) as the enrollment utterance and explored the distribution of EnW when utterance2 and utterance3 from speaker A (denoted as SpkA_utt2 and SpkA_utt3, respectively), and utterance4 from speaker B (denoted as SpkB_utt4) are used as the evaluation utterance. As shown in Figure 7, the horizontal axis represents the index of frames and the vertical axis represents the corresponding coefficient of EnWs. The EnWs follow a similar distribution when the evaluation utterances are from the same speaker. For instance, the EnW corresponding to SpkA_utt2 is highly correlated with that corresponding to SpkA_utt3. However, similar to human selective attention [29], the EnWs differ greatly when the evaluation utterances are from different speakers. For instance, the BaCNN model payed more attention to the first 58 frames of SpkA_utt1 when comparing it with SpkA_utt2, whereas it payed more attention to the first 35 frames of SpkA_utt1 when comparing with SpkB_utt4. The attention weights for the enrollment utterances varied with evaluated speakers. This also indicates that the BaCNN model does learn interactive speaker representations for different speakers.

## 6. Conclusions

Inspired by the selective auditory attention of human brain, we were motivated to design a novel bidirectional attention mechanism for text-dependent speaker verification. Specifically, we investigated a CNN-based network used to extract frame-level hidden features, since it has been proven to be effective in speaker verification. The literature demonstrates that the emerging TDSV methods always neglect the joint information between the enrollment and evaluation utterances. Instead of using a fixed enrollment speaker representation in speaker verification, we employed a bidirectional attention mechanism to model the interactive speaker representations in comparing with the utterances from different speakers. Considering the complementary characters, we combined the interactive information and the constant hidden features in calculating the similarity between enrollment and evaluation utterances. In view of the importance of lexical contents in TDSV, we introduced an additional loss to jointly explore the speaker-discriminant and speech-discriminant information. Experimental results on the internal “Dan Dan Ni Hao” benchmark demonstrated a significant improvement of BaCNN against various baselines, including i-vector/PLDA, d-vector, self-attention, and Seq2seq attention. The proposed BaCNN mimics the human selective auditory attention, and therefore can also be applied to text-independent speaker verification tasks.

## Figures and Tables

**Table 1:** The EERs (%) on the development and test sets with and without $L_{NET1}$.

Losses	Development Set	Test Set
$L_{NET2}+L_{NET4}$	6.60	6.62
$5L_{NET1}+L_{NET2}+L_{NET4}$	6.27	6.26

**Table 2:** The EERs (%) on the development and test sets with different losses.

Architecture	Losses	Development Set	Test Set
d-vector	CEloss+tripletloss	8.03	7.99
d-vector	CEloss+circleloss	7.43	7.18
BaCNN	$0.5L_{NET1}+L_{NET2}\left(CE\;loss+triplet\;loss\right)+0.5L_{NET4}$	6.60	6.51
BaCNN	$5L_{NET1}+L_{NET2}\left(CE\;loss+circle\;loss\right)+L_{NET4}$	6.27	6.26

**Table 3:** The EERs (%) on the development and test sets with different inputs.

Inputs of NET4	Development Set	Test Set
EnH and EvH	6.55	6.62
EnR and EvR	7.55	7.18
EnH, EvH, and EnR	6.25	6.41
EnH, EvH, and EvR	6.33	6.58
EnH, EvH, EnR and EvR	6.27	6.26

**Table 4:** Comparison with state-of-the-art methods on the development set and the test set.

Method	Development Set			Test Set		
	EERR (%)	${\mathrm{Recall}}_{0.05}$ (%)	${\mathrm{MinDCF}}_{0.01}$	EER (%)	${\mathrm{Recall}}_{0.05}$ (%)	${\mathrm{MinDCF}}_{0.01}$
i-vector/PLDA [12]	11.61	77.51	0.5578	11.80	76.83	0.5499
d-vector and cosine [25]	7.43	87.67	0.4033	7.18	89.42	0.4017
Self-attention [19]	6.96	90.40	0.3795	6.87	89.98	0.4235
Seq2Seq attention [7]	6.88	89.57	4059	6.83	89.73	0.4236
BaCNN-1step	7.60	88.10	0.4373	6.91	89.18	0.4606
BaCNN	6.27	92.00	0.3709	6.26	91.42	0.3996
